# Network Characteristics of Successful Performance in Association Football. A Study on the UEFA Champions League

**DOI:** 10.3389/fpsyg.2017.01173

**Published:** 2017-07-13

**Authors:** Tiago J. Pina, Ana Paulo, Duarte Araújo

**Affiliations:** Interdisciplinary Centre of Human Performance (CIPER), Faculdade de Motricidade Humana, Universidade de Lisboa Lisbon, Portugal

**Keywords:** Social Network Analysis, team sports, elite soccer, match analysis, expert performance, team synergy

## Abstract

The synergistic interaction between teammates in association football has properties that can be captured by Social Network Analysis (SNA). The analysis of networks formed by team players passing a ball in a match shows that team success is correlated with high network density and clustering coefficient, as well as with reduced network centralization. However, oversimplification needs to be avoided, as network metrics events associated with success should not be considered equally to those that are not. In the present study, we investigated whether network density, clustering coefficient and centralization can predict successful or unsuccessful team performance. We analyzed 12 games of the Group Stage of UEFA Champions League 2015/2016 Group C by using public records from TV broadcasts. Notational analyses were performed to categorize attacking sequences as successful or unsuccessful, and to collect data on the ball-passing networks. The network metrics were then computed. A hierarchical logistic-regression model was used to predict the successfulness of the offensive plays from network density, clustering coefficient and centralization, after controlling for the effect of total passes on successfulness of offensive plays. Results confirmed the independent effect of network metrics. Density, but not clustering coefficient or centralization, was a significant predictor of the successfulness of offensive plays. We found a negative relation between density and successfulness of offensive plays. However, reduced density was associated with a higher number of offensive plays, albeit mostly unsuccessful. Conversely, high density was associated with a lower number of successful offensive plays (SOPs), but also with overall fewer offensive plays and “ball possession losses” before the attacking team entered the finishing zone. Independent SNA of team performance is important to minimize the limitations of oversimplifying effective team synergies.

## Introduction

The team, rather than the individual, has become the basic work unit in many activities and organizations (Balkundi and Harrison, 2006), and team sports are excellent examples revealing the importance of team dynamics for success (Duch et al., 2010). A team is a group of individuals working cooperatively and in a coordinated way to achieve a common goal (Zaccaro et al., 2002). Team performance is more than the sum of the interdependent individual performances, as individuals strive to coordinate between different roles and tasks (Anderson and Franks, 2001). In team sports performance, individual players in a successful team act as a coherent unit, thus creating a team synergy (Araújo and Davids, 2016).

Individual and collective behavior has been intensively studied in team sports performance analysis. The behavior of an individual player affects the team's behavioral pattern (Vilar et al., 2012), and conversely, the teammates may influence the behavior of each individual player. Team behavior is a collective organization that emerges from the cooperation between teammates (Gréhaigne et al., 1997; Peña and Touchette, 2012). The emergence of such collective behaviors can be assessed and understood through the measurement of key synergistic properties such as degeneracy, i.e., the structurally different components that perform a similar (but not necessarily identical) function in a given context (Araújo and Davids, 2016). The degeneracy of team behavior as a social relationship property can be captured by Social Network Analysis (SNA) (Grund, 2012; Peña and Touchette, 2012). SNA has been applied to association football or soccer (Clemente et al., 2014b), in particular to analyze ball-passing networks in a team. These studies demonstrated that some metrics are useful to characterize styles of play and cooperation among teammates (Cotta et al., 2011, as well as the relation between individual actions and team tactical behavior (Passos et al., 2011). Centrality metrics have been used to identify the most influential tactical positions within a team. For example, by analyzing the in-degree and out-degree centrality of the Portugal national football team players, Mendes et al. (2015) found that during the FIFA World Cup 2014 the central midfielders were the key players in the attacking-building process. A similar study examining degree centrality and degree prestige of Switzerland national team players during the same competition showed that the key players receiving the ball were also the midfielders, suggesting this team has a style of play based on attacking building (Clemente et al., 2015b). Thus, network metrics such as density, heterogeneity and centralization are effective for characterizing the cooperation between players (Clemente et al., 2015a).

Analyses of network heterogeneity and centrality reveal that team offensive play has many variations and short patterns that increase collective unpredictability (Clemente et al., 2014b). Furthermore, high total links and high density can convey the team's greater ability to pass the ball between all players and to function as a whole, as well as to decentralize the network (Clemente et al., 2014a). For example, a study analyzing team ball-passing networks in 760 matches of the English Premier League (Grund, 2012) showed that high levels of network intensity were associated with increased team performance (goals scored), and centralized interaction patterns with decreased team performance. More recently, similar research analyzing ball-passing networks of teams competing at the FIFA World Cup 2014 (Clemente et al., 2015c) revealed significant differences in density, total links and clustering coefficient between teams reaching different stages of the competition. These findings further demonstrate an association between higher density, total links and clustering coefficient with performance variables such as goals scored, overall shots, and shots on goal (Clemente et al., 2015c). These findings were corroborated in youth football (under-15 and under-17) by Gonçalves et al. (2017), who observed that lower passing dependency for a given player (lower betweenness scores) and higher intra-team well-connected passing relations (higher passing density and closeness scores) may optimize team performance (number of shots). Also outside the scope of SNA important contributions were made to understand the effectiveness of collective behaviors and different tactical approaches. Thus, longer passing sequences, either in terms of number of passes (Hughes and Franks, 2005; Tenga et al., 2010a) or its duration (Lago-Ballesteros et al., 2012a) have been reported as more efficient to obtain goals (Hughes and Franks, 2005) or score-box possessions (Tenga et al., 2010a; Lago-Ballesteros et al., 2012a).

Despite these recent advances, research in the field has remained focused on the association between ball-passing network metrics and coarse-grained team performance variables (e.g., goals scored, shots, shots on goal, or competition stage reached) (Grund, 2012; Clemente et al., 2015c), which implies that team performance outputs and network properties metrics are measured simultaneously (Grund, 2012). However, since ball-passing network analysis offers an overall picture of events occurring during a certain period of time, typically a synthesis of several complete matches, the events leading to successful or unsuccessful team performance are included in the same analyses. Thus, it remains unknown whether specific network properties and successful (or unsuccessful) team behavior are associated. Furthermore, although previous research based on ball-passing networks suggests that high density (Clemente et al., 2015c) and low centralization (Grund, 2012) are associated with successful teams, the relation between clustering coefficients and team performance is more uncertain (Peña and Touchette, 2012; Gudmundsson and Horton, 2016). Thus, the aim of this study was to test whether team network density, centralization and clustering coefficient can be used to predict the outcome of offensive plays.

## Materials and methods

### Sample

This study deliberately focused on club-teams rather than on national teams because club-teams train and compete together for longer consecutive periods of time. Our sample comprises 12 matches played in Group C of the UEFA Champions League 2015/2016 Group Stage. The four teams analyzed are here identified as CAM, FCA, GSK, and SLB.

### Procedures

Our analysis focused on collective offensive processes. Offensive play is a set of attacking actions performed by a team between recovering and losing ball possession. According to Garganta (1997) a team is in possession of the ball, and therefore in the attacking process, when any of its players respect, at least, one of the following conditions: (i) holds at least two consecutive contacts with ball, (ii) performs a positive pass (allowing the maintenance of ball possession), and (iii) performs a shot (finishing). We considered that a team is in possession of the ball once it completes a pass and maintains ball possession after the pass. Moreover, set-off passes were considered in the analysis.

The video footage used in the analysis was obtained from TV broadcasters. We started by categorizing all offensive plays as *successful* when the attacking team entered the *finishing zone*, which was previously reported as a proxy variable for scored goals when measuring successfulness in football (Tenga et al., 2010b). The concept of finishing zone was based on Gréhaigne et al.'s longitudinal division of the football field into four equal areas (Gréhaigne et al., 2001). These areas are designated according to the direction of the attack as follows: defensive zone, pre-defensive zone, pre-offensive zone and offensive zone. The offensive zone in elite soccer was defined as the finishing zone (Lago Ballesteros et al., 2012b).

*Successful offensive plays* (SOPs) include plays that finished with a shot at the goal and those where the team retained ball possession until entering the finishing zone. *Unsuccessful offensive plays* (UOPs) were all the plays where the team lost ball possession without meeting either of the SOP criteria. *Neutral plays* were offensive plays where a team did not lose ball possession but also did not meet the SOP criteria. This neutral category included all offensive plays that were initiated: (i) from an offensive corner kick; (ii) in an offensive throw-in; and (iii) from offensive free kicks with a first pass directly into the finishing zone. The neutral offensive plays were not included in the present analysis.

The offensive plays were identified and categorized with *Longomatch* software from every pass performed in the 12 matches. The players who passed and received the ball were registered for each offensive play. A number from one to 11 was assigned to each player according to his initial position within the team's tactical system. The same number was assigned to players performing the same tactical position. Taking into account their different stoppage times, each half of the match was divided into three fractions with the same duration. Next, two adjacency matrices of offensive plays (successful and unsuccessful) for each opposing team were created for the six periods of the match, in a total of 24 adjacency matrices per match. Each of these adjacency matrices was then imported to the software *NodeXL* to compute the networks and their metrics. All statistical procedures were performed using *SPSS Statistics 24*.

### Predictor variables

#### Density

Density is the interconnectedness of nodes (players) in a network (team), i.e., it is the ratio of existing ties (passes) between teammates relative to the possible number of such ties (Balkundi and Harrison, 2006). In ordered relations, as in the teammates interactions, the possible directed links in a digraph of n nodes are *n* (*n* − 1), as a unique pass between two players was operationally defined as a link. The graph's density Δ is defined as the ratio between the total registered links (L) and the maximum number of possible connections. It is calculated as:

$$\begin{array}{l} \Delta =\frac{L}{n\;\left(n-1\right)} \end{array}$$

Thus, density is a fraction with a minimum of 0 (no lines/arcs present) and a maximum of 1 (all lines/arcs are present) (Wasserman and Faust, 1994).

#### Clustering coefficient

Clustering is a measure of the degree to which nodes in a network tend to cluster together (Peña and Touchette, 2012). The clustering coefficient, originally introduced by Watts and Strogatz (1998), quantifies how close a node and its neighbors in a graph are to becoming a complete subgraph.

In directed graphs, the local clustering coefficient of a vertex expresses the ratio of the links between the vertices that are connected to it. Thus, local clustering coefficient (C) of a given vertex *i* is the fraction of the number of connections *a*_*jk*_ between *k*_*i*_ vertices in its neighborhood, divided by the maximum number *k*_*i*_ (*k*_*i*_ − 1) of possible links there between:

$$\begin{array}{l} C_{i}=\frac{\left|\left\{a_{jk},a_{jk}\in E\right\}\right|}{k_{i}\left(k_{i}-1\right)} \end{array}$$

We used a variant of the clustering coefficient—the average local clustering coefficient—to measure the clustering level throughout the network:

$$\begin{array}{l} \overset{-}{C}=\frac{1}{n}\sum_{i=1}^{n}C_{i} \end{array}$$

#### Centralization

The centrality of a group or network is the degree of inequality of the distribution of positions/“weights” of different elements within the network. A network is therefore more centralized when one of its elements is clearly more central than all other group members. Conversely, a network is decentralized when all its elements have the same value of centrality (Grund, 2012).

There are several measures of centrality and researchers do not always agree on how “group centrality” or “centralization” should be assessed. We used degree centrality for quantifying the relative influence of each player on the total number of passes within a network. Thus, centralization conveys how central the most central player is when compared to the other players in the network. This metric was originally described by Freeman (1978) and is calculated as the sum of the differences between the vertex with the highest degree centrality and all other vertexes; divided by a value depending only on the size of the network:

$$\begin{array}{l} C_{D}=\frac{\sum_{i=1}^{n}\deg\left(v^{*}\right)-\deg\left(v\right)}{n^{2}-3n+2} \end{array}$$

where deg (v^*^) is the largest value of centrality degree in the network, deg (*v*) is the value of each vertex centrality degree, and the denominator is the maximum possible sum of differences in *i* = 1 vertex centrality for a graph of *n* vertexes (Freeman, 1978).

In the context of a football match, zero centralization indicates that all players have the same level of interaction during the game. Conversely, a centralization value very close to one suggests that a player is the key-player of the team and that other players have a strong tendency to play with him (Clemente et al., 2015a).

### Analysis

A hierarchical logistic regression model using the logit link function was performed to predict the successfulness of offensive plays from the number of passes performed and the network metrics (density, clustering coefficient and centralization). Two blocks were defined. In thefirst block, only the predictor *total passes* was introduced. In the second block, we introduced the network metrics. Thus, after controlling for the effect of total passes, we could estimate the specific effects of the network metrics. Preliminarily, the data was screened for collinearity problems and outliers and for linearity of the logit. Following the recommendations in (Belsley et al., 2005), we diagnosed collinearity when conditioning indexes were greater than 30 for a given dimension and the variance proportions were greater than 0.5 for more than one variable. The latter was true for the pairs of variables “clustering coefficient and centralization” and “total passes and density,” however, both of these dimensions registered conditioning indexes below 30 (12.224 and 22.655, respectively). We tested all the metrics for linearity of the logit, running the logistic regression with all predictors and the interaction between each predictor and the log of itself in a single block. All four interactions had significance values greater than 0.05, indicating that the assumption of linearity of the logit has been met for total passes, density, clustering coefficient and centralization. Consequently, it was not necessary to transform or eliminate any predictor-variable. Next, we obtained *z*-scores and searched for outliers greater than 3.29 (Tabachnick and Fidell, 2013). A single outlier was identified (z-score = 4.378) and removed. Additionally, four SOP cases were removed because they registered “no passes.” After these preliminary procedures, 283 of the initial 288 cases were kept for further analysis, corresponding to 144 cases of UOP and 139 of SOP.

In a logistic regression, Exp (β_*i*_) represents the odds-ratio of success vs. failure (categories of the model's dependent variable) when variable *X*_*i*_ increases by one unit with respect to the odds-ratio of success vs. failure, when *X*_*i*_ stays constant. Density, clustering coefficient and centralization vary between zero and one, therefore, we converted these metrics to a scale of 0 to 10 to adjust to model sensitivity. Consequently, the odds ratios presented for these variables refer to a unit change of 0.1.

## Results

A two-block hierarchical logistic regression was used to predict the successfulness of offensive plays. In the first block, the total number of passes (hereafter referred to as ‘total passes’) was the only predictor-variable. This model performed significantly better than a constant-only model [$G_{\left(1,\;N=283\right)}^{2}=7.484,\;p=$ 0.006], it did not satisfy goodness-of-fit criteria (Hosmer and Lemeshow test: $\chi_{\left(8,\;N=283\right)}^{2}$ = 25.342, *p* = 0.001), and it produced a Nagelkerke *r*^2^ of 0.035. Network metrics were added in a second block (Table 1). This second model performed better than a constant-only model [$G_{\left(1,\;N=283\right)}^{2}=15.484,p=$ 0.004) and satisfied goodness-of-fit criteria (Hosmer and Lemeshow test: $\chi_{\left(8,\;N=283\right)}^{2}$ = 7.187, *p* = 0.517), achieving a Nagelkerke *r*^2^ of 0.071. The first-block model correctly classified 56.2% of the known cases, 66.7% of the UOPs and 45.3% of the SOPs. The second-block model correctly classified 69.5% of the UOPs and 47.5% of the SOPs, with an overall correct classification of 58.7% of the cases. Thus, adding the second block to the model increased the number of correct classifications by 2.5%.

**Table 1:** Binary Logistic Regression Model of offensive plays' successfulness.

	**β (S.E.)**	**Wald**	***p***	**Exp (β)**	**Exp (β) 95% C.I**	
					**Lower**	**Upper**
Total number of passes	0.079 (0.034)	5.475	0.019	1.082	1.013	1.156
Density scores	–1.320 (0.591)	4.994	0.025	0.267	0.084	0.850
Clustering coefficient scores	0.179 (0.193)	0.858	0.354	1.196	0.819	1.747
Centralization scores	0.189 (0.143)	1.759	0.185	1.208	0.914	1.597
Constant	–0.615 (0.469)	1.719	0.190	0.541		

*Successful Offensive Play (SOP) is the reference category of successfulness predicted in the model*.

**Figure 1:**
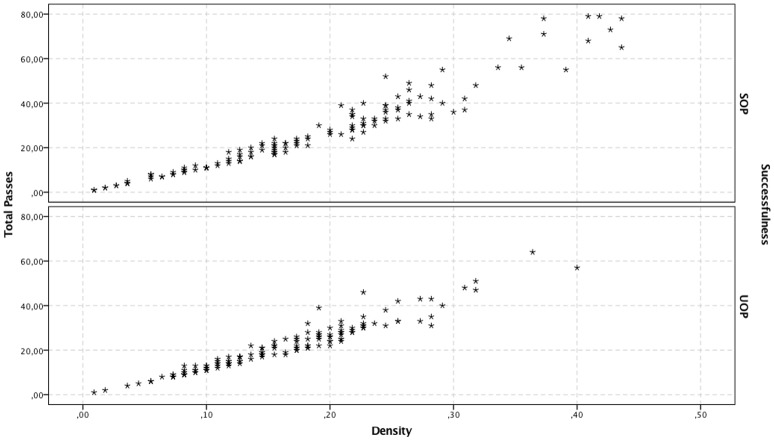
Depiction case-by-case of the relationship between density and total passes, for SOP and UOP predicted outcomes, according to the second-block logistic regression model.

**Figure 2:**
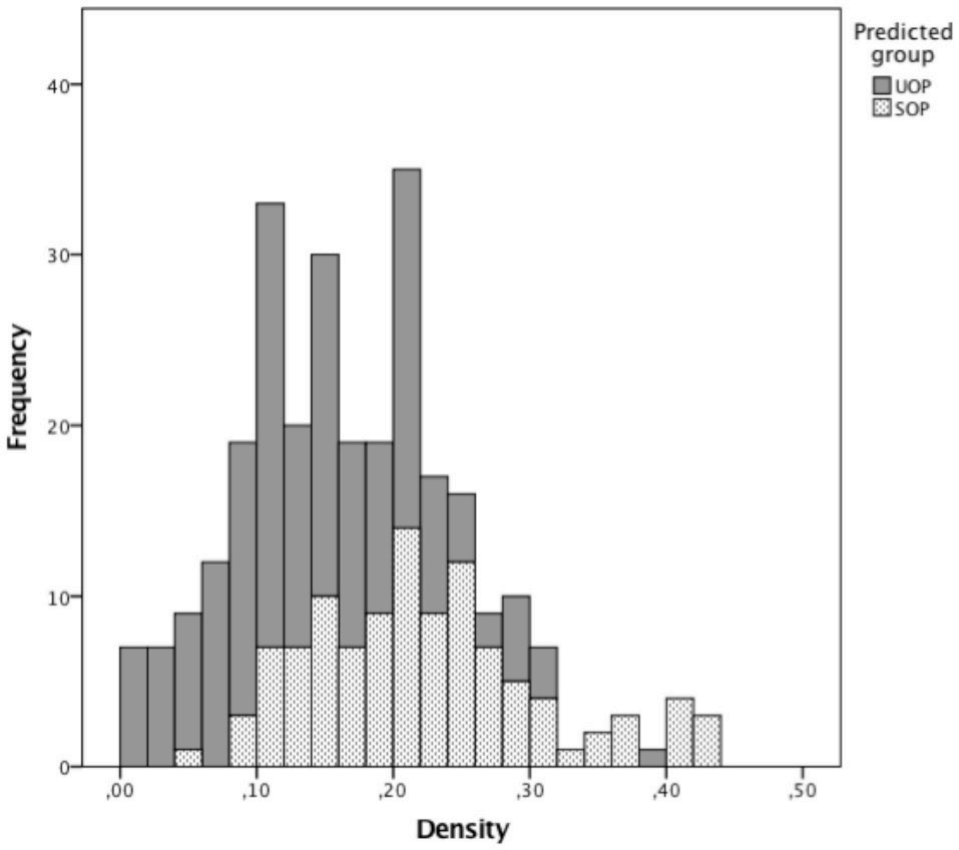
Frequencies of density values, according to the category of offensive play's successfulness.

Total number of passes and density were significant predictors among the four considered variables. The total number of passes was positively associated with the successfulness of offensive plays. A one-pass-increase augmented the probability of SOPs by 8.2% Exp (β) = 1.082; see Table 1). More significantly, a 10% decrease in density increased the chances for a successful offensive play by 73.3% (Exp (β) = 0.267; see Table 1). Furthermore, for density values ranging from 0 to 0.25 there is a similar relation between total passes and number of either SOPs or UOPs (see Figure 1), despite the higher frequency of UOPs (see Figure 2). However, for density values above 0.25, as density and total passes increases, we see a tendency for a decrease in both SOPs and UOPs, but a predominant occurrence of SOPs in relation to UOPs.

## Discussion

Network characteristics such as density, clustering coefficient and centralization have been reported as good descriptors of game style in soccer teams, as they can be associated with metrics of success such as goals scored, shots, shots on goal, and competition stage reached by teams. However, since network analysis describes events occurring during entire matches, performance outputs and network properties metrics cannot be measured simultaneously. In this study, we attempted to clarify the association between specific network properties and successful (or unsuccessful) team behavior.

Our model was able to classify 58.7% of the events correctly, however, it performed better at identifying UOPs (69.5%) than SOPs (47.5%). These results suggest that these network metrics (density, clustering coefficient and centralization) can more accurately describe the team behaviors associated with UOPs (i.e., losing ball possession) than the behaviors leading to SOPs (i.e., moving into the finishing zone or shooting on goal). Thus, despite the limited predictive power, the model seems to better pinpoint the collective behaviors that the teams should avoid rather than the ones that they should perform in order to ensure success.

The total number of passes and density were the most relevant variables in our model. Total passes was introduced in the first block of regression model to assess the specific influence of the network metrics on team performance. The improvement in the model obtained by adding the second block confirmed the metrics' specific influence. We observed a positive association between total passes and team performance. Each new pass in a set of offensive plays occurring within a 15 min-period resulted in the teams being 8.2% more likely to move into the finishing zone or to shoot on goal. These findings corroborated the studies that showed that long passing sequences are more efficient than short passing sequences (Hughes and Franks, 2005; Tenga et al., 2010a; Lago-Ballesteros et al., 2012a). The density of a ball-passing network increases whenever two players who were not yet connected pass the ball between them; in this way, high density is probably associated to high occurrence of these differentiated links. This greater variability of pass patterns, which is expressed in qualitatively distinct connections over a given period, may occur for different reasons. For example, greater collective dynamics and high player mobility can result in passes between players who regularly play in distant areas.

It has been shown that strong cooperation between teammates makes teams stronger and more successful (Balkundi and Harrison, 2006). Thus, how can we explain our results showing that density has a negative effect (albeit small) on the successfulness of offensive plays? As can be seen in Figure 2, for density values ranging from 0 to 0.25 our model predicts more UOP than SOP outcomes. When we consider only events classified as SOP, there is a high number of offensive plays with density values ranging from 0.1 to 0.25, followed by a decrease. This drop in the number of offensive plays for higher density values could explain the negative association between density and SOPs. Indeed, despite being associated with fewer SOPs overall, higher densities are more likely to lead to SOPs (see Figure 1). Thus, our results suggest that density values lower than 0.25 are associated with a higher number of offensive plays, albeit mostly unsuccessful ones. Conversely, for density values above 0.25 there may be fewer offensive plays overall but most are successful. It is unlikely though that this negative association between density and SOPs is simply due to the higher number of errors and losses that result from the players' greater efforts to maintain connections in high-density scenarios (Burt, 1997). Instead, it seems more plausible that the reduction in SOP outcomes observed for density values above 0.25 explains that negative association. Indeed, these offensive plays with high-density values are characterized by a higher number of passes (see Figure 1), which could explain why there are fewer (but more successful) offensive plays in the same period of time. For example, these high-density values may result from longer ball-possession times, fewer ball possession losses, or specific losses in advanced zones of the field (finishing zone). These results are in line with findings of Hughes and Franks (2005), who reported that the association between short offensive sequences and high number of goals was directly related to the greater number of these sequences but not to their efficiency. When the results were normalized by the number of offensive plays, it was observed that the longer offensive plays were more efficient. This hypothesis is consistent with our observation that qualitatively differentiated links are associated with high densities, which likely reflects a greater unpredictability of passing patterns. Furthermore, it was previously proposed that greater variability of action and less exposure to the opponent could result from decentralized passing patterns (Gréhaigne et al., 1997). Such characteristics of offensive plays associated with high-density values contribute to an offensive process that creates goal-scoring opportunities and are more effective for maintaining ball possession in advanced areas. Interestingly, offensive plays with similar characteristics have been observed in successful teams at the FIFA World Cup 2014 (Clemente et al., 2015c) and in under-15 and under-17 football teams (Gonçalves et al., 2017).

We found that the clustering coefficient is not a significant predictor of the successfulness of offensive plays, thus corroborating previous research (Peña and Touchette, 2012; Gudmundsson and Horton, 2016). High clustering coefficient values express the subgroup formation within the team itself; when these subgroups are created based on passes between teammates, as in the present study, the players performing in close areas tend to be linked together, thereby explaining the high clustering coefficients. This could reflect an offensive style choice based on short combinations between players, as previously observed for the Spain, Germany and Netherlands national teams at the FIFA World Cup 2010 (Cotta et al., 2011; Peña and Touchette, 2012). Thus, the modest contribution of the clustering coefficient to the predictive value of our model suggests that different offensive styles may lead to successful team performance, depending, for example, on the players' individual qualities or on different strategic options. Further investigation is needed to clarify this issue. Our results also demonstrated that centralization is not consistently associated with successfulness of offensive plays, which is in agreement with findings by Fewell et al. (2012) showing that there is no strong relationship between centralization and team performance. Results didn't corroborate previous reports showing that higher centralization is associated with worse team performance (Grund, 2012; Gonçalves et al., 2017). This discrepancy could, however, be explained by the different methodologies in these studies, as discriminating successful and unsuccessful performances probably influenced the relationship between centralization and successful team performance in our study.

In summary, our results suggest that network density contributes to the prediction of a team's ability to enter in the finishing zone or to shoot at the goal in elite football matches. Furthermore, this study gives new insights into the association between network density and team performance (Balkundi and Harrison, 2006). First, we showed that low network density may be associated with a higher overall number of offensive plays but which are mostly unsuccessful. Second, high density was associated with fewer and/or longer offensive plays, which reduces the possibilities of a team moving into the finishing zone (hence decreasing total SOPs), thus resulting in a negative association between density and SOPs. Finally, we considered that high density may also be associated with fewer ball-possession losses before the teams reach the finishing zone (hence increasing probability of SOPs), thereby supporting the density-performance hypothesis.

Some practical implications can be drawn from the present findings. Teams that express high densities in their offensive process may lose possession of the ball in the advanced zones, This facilitates, for example, more space on the back of the defensive line and the need to control this space by efficient pressing in zones of loss. Furthermore, the establishment of varied links by a team is eventually dependent on the creation of numerous lines of pass to the player with the ball. In light with ecological dynamics (Araujo et al., 2006), it might be enhanced in the training sessions by the manipulation of task constraints, such as: (i) using different relationships between depth/width of field, to make a team enter the finishing zone by different space channels and, consequently, using differentiated links; (ii) performing possession games with numerous mini-goals dispersed in the field, so that the player with the ball searches for 360° pass lines (all around him/her); (iii) performing games with variation of the relationship between the number of players and the size of the field, to induce variability in the distance of the pass lines and the type of pass required. On the other hand, teams that express less density in their offensive plays must be prepared for more losses of ball possession, most probably in areas closer to their goal. In addition, to be offensively successful with more constant links among teammates (less new links), maybe some useful task constraints might be: (i) establishment of a time limit for the performance of offensive plays, in order to enhance the entries in the finishing zones with few connections; (ii) performing small-sided games with few players (1 × 1, 2 × 2, 3 × 3) to promote brief attacking actions with stable connections; (iii) improving relationships between specific players, according to preferential links, by placing such players in the same team in small-sided games or in the training of specific collective actions among them.

We tested a model that analyzes the specific associations between the characteristics of a team's ball-passing network and the outcome of its offensive plays (entering the finishing zone and shot on goal vs. losing ball possession). Previous studies had not differentiated these different outcomes, which may explain our results revealing a negative relation between density and team performance. Additionally the limited predictive power of the model may be associated with some limitations of the study such as the reduced number of teams and games analyzed, which may influence the findings due to the specific style of play of the four teams and eventually by the intra- and inter-team synergies created in the matches among them. Finally, we demonstrated that neither clustering coefficient nor centralization are significant predictors of team performance successfulness, possibly indicating that diverse offensive styles can be equally effective for a team to succeed.

## Author contributions

TP had a major contribution to study conception and design, acquisition of data and analysis and interpretation of data. AP had a major contribution to analysis and interpretation of data. DA had a major contribution to study conception and design and analysis and interpretation of data.

### Conflict of interest statement

The authors declare that the research was conducted in the absence of any commercial or financial relationships that could be construed as a potential conflict of interest.
